# Comparison of the Clusters and Non-Clusters Areas of Attempted Suicide Cases in Hamadan Province, Western Iran: Findings from a Pilot Study (2016-2017)

**Published:** 2018-08-20

**Authors:** Manoochehr Karami, Saeid Yazdi-Ravandi, Ali Ghaleiha, Meysam Olfatifar

**Affiliations:** ^1^ Modeling of Noncommunicable Diseases Research Center, Hamadan University of Medical Sciences, Hamadan, Iran; ^2^ Behavioral Disorders and Substance Abuse Research Center, Hamadan University of Medical Sciences, Hamadan, Iran; ^3^ Young Researchers and Elite Club, Rudehen Branch, Islamic Azad University, Rudehen, Iran; ^4^ Gastroenterology and Liver Disease Research Center, Research Institute for Gastroenterology and Liver Disease, Shahid Beheshti University of Medical Sciences, Tehran, Iran; ^5^ Student Research Committee, Hamadan University of Medical Sciences, Hamadan, Iran

**Keywords:** Suicide, Spatial analysis, Injury, Epidemiology, Iran

## Abstract

**Background:** Suicide behaviors are complex and multifactorial problems that in the most of the societies are considered as the public health challenge. However, its underlying reasons and spatial pattern remain unclear in Hamadan Province, western Iran.

**Study design:** Secondary analysis of existing data.

**Methods:** We assessed the spatial pattern pre-city regarding some influencing factors by scan-statistics and logistic regression to detect clusters areas and its comparison with other areas for the period of 2016- 2017. All of the registered cases of attempted suicide in a quality registry system of suicide in Sina (Farshchian) Hospital affiliated to Hamadan University of Medical Sciences, Hamadan, Iran were enrolled.

**Results:** Two significant clusters were detected in study areas, formed with relative risk at 5.28 (*P*=0.001) and 6.33 (*P*=0.017), and with the centrality of Asadabad and Razan, respectively. Clusters and nonclusters areas were differed in terms of location (OR=0.15, 95%, CI: 0.07, 0.31), self-harms methods (OR=0.28, 95%, CI: 0.9, 0.88) and education. Residents of rural areas, illiterate people and non-drug user cases have more likely to be in a cluster.

**Conclusions:** Clusters were not formed equally among cities of Hamadan Province. Accordingly, we suggest the implementation of appropriate, long-term and evidence-based educations for high-risk and vulnerable groups through the intersectoral interventions in different parts of Hamadan Province (considering the cluster and non-clusters areas) to avert deaths and related injuries from attempted suicide.

## Introduction

Attempted suicide behaviors refer to self-poisoning or self-injury, regardless of motive or intention scope to commit suicide ^1^. The prevalence of suicide behaviors in Iran is lower than the global statistic, but it remains as public health problems now, and are responsible for a large proportion of disability-adjusted life years (DALYs) in Iran and world ^2^ as well 200 years of life lost (YLL) per 100000 people in Iran ^3^.

Reducing the suicide rate is a key priority for most of the communities. One of the clinical and public strategies for this purpose is the reinforcement of the health and psychological cares for high-risk populations^4^. However, characteristic of the suicide and its related behaviors are different based on age, sex, socioeconomic and other influencing variables not only across countries as shown in Florida^5^, Australia^6^, United States^7^, and Iran^8^; but also across regions of a country^9^ and change over time^10^. Therefore, the study of the suicidal behaviors during time periods and different regions, even in one country is more crucial to the implementation of evidence-based suicide prevention and control initiatives.

There are several reports used spatial analysis methods to detect high-risk areas (clusters) of suicide^11-13^ because it obtains clues to detect the diseases, injuries and mortality causes and also planning to provide appropriate intervention in different parts of a country^14^ to avert deaths and related injuries from attempted suicide, therefore we used these techniques to delineate self-harm cases clusters and subsequent regression analysis to assess the impact of underlying variables on these clusters. We determined two significant clusters of self-harms cases in Hamadan Province that might indicate the need to urgently intervene to prevent more contagion of these behaviors.

## Methods

This spatial analysis study examined attempted suicide clusters in Hamadan, western Iran from Oct 2016 to Feb 2017. Suicide data were obtained from a quality registry system of suicide in Sina (Farshchian) Hospital affiliated to Hamadan University of Medical Sciences, Hamadan, Iran. After removing the incomplete and cases belong to other neighborhood provinces such as Kermanshah, analysis was done on 402 remaining data (18 cases were removed). The following information for each included cases was obtained: age, sex, marital status, methods of suicide, residential and some other demographic and additional information. A case was considered to be committed suicide who was actively seeking to harm himself, or if he was injured persistently or permanently.

To detect suicides spatial clusters, the scan statistic method was used. In summary, SaTScan software imposes a search window on coordinate center assigned to unites (cities) in study areas and with calculating the likelihood ratio based on the following formula and compares to the amount achieved on the Monte Carlo simulation to reject the null hypothesis, means that rates inside the window are equal to rates outside of it.

$$I={\left(\frac{c}{EI(c)}\right)}^{c}{\left(\frac{C-c}{C-E(c)}\right)}^{C-c}$$

In this equation, C is the total number of cases, c the observed cases inside widow, E(c) the expected number of cases inside the window, C-c and C-E(c) are the observed and expected number of cases outside of the window. We used Geographic Information System (GIS) to determine unites (Hamadan cities) coordinate center and depiction the SaTScan output results. Then, logistic regression analysis and Pearson’s chi-square were used to more investigation of the clusters areas and compare them with other areas. In such a way, people belong to the identified clusters units were considered as cases and people lived (belong) in other areas were considered as controls, and in each case, the impact of age, sex, place of residence (city/village), literacy, occupation, and method of suicide variables was investigated. In all situation, the significant level was considered at (*P*<0.05). The Research Ethics Committee of Hamadan University of medical sciences approved this study.

## Results

### Spatial analysis

**Figure 1:**
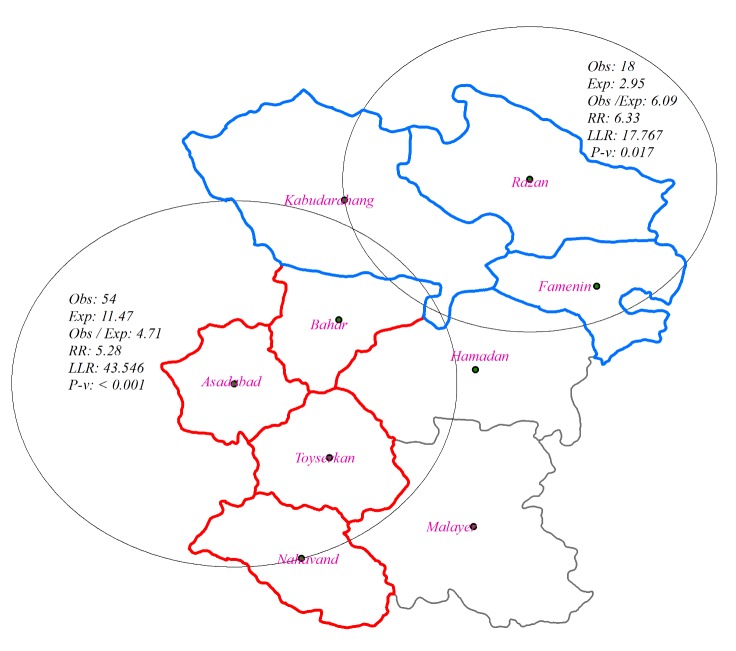
Geographical locations of suicide attempt clusters in Hamadan Province: red and blue cities are compositing unites of the first and second clusters respectively

Considerable evidence indicate that spatial analysis can demonstrate the prioritization of interventions in high-risk areas. We used scan statistic to determine spatial clusters of suicide in Hamadan Province. Two primary clusters were detected which totally comprised 72 cases out of 402 cases. The first cluster with the centrality of Asadabad City, with the radius of 64.99 km and relative risk of 5.28 was formed of Asadabad, Toyserkan, Bahar and Nahavand cities (*P*<0.001) (Figure 1). The second cluster with the centrality of Razan, with the radius of 54.19 km and relative risk of 6.33 was composed of Razan, Famenain, and Kabudarahang (*P*<0.017) (Figure 1). These results suggest that probably Razan, Famenain, and Kabudarahang cities are high-risk areas of attempted suicide in Hamadan Province.

### Comparison of the cluster and non-clusters areas

Clusters areas had some potential differences compared to non-clusters areas ^15^. We used regression analysis and Pearson’s chi-square to evince of these underlying differences. Cluster and non-clusters suicide differed in their location (OR=0.15, CI: 0.07,0.31) meaning that the odd of cluster formation in urban areas was 0.152 less than rural areas. Suicide by drug user people was also low odd to be in a cluster (OR=0.28, CI: 0.9-0.88). Finally, more educated people had low odds to occur in clusters (OR=0.33, CI: 0.14-0.76 and OR=0.30, CI: 0.10-0.86). In particular, rural (36.11% vs. 8.16%, *P*=0.000), illiterate (15.49% vs. 5.68%, *P*=0.017) and non-drug user had more likely to be in a cluster (Table 1). These results partially approved the potential differences between clusters and non-clusters areas.

**Table 1:** Descriptive and analytic characteristic of clustered (n=72) and non- clustered (n=331) self-harms cases

**Variables**	**Clustered** **Number (%)**	**Non-clustered** **Number (%)**	**Unadjusted** **OR (95% CI)**	***P*** ** value**	**Adjusted** **OR (95% CI)**	***P*** ** value**
Sex						
Male	150 (45.3)	32 (44.4)	1.00		1.00	
Female	181 (54.6)	40 (55.5)	1.03 (0.62,1.72)	0.893	1.06 (0.50, 2.24)	0.825
Age(yr)						
<25	17 (23.6)	60 (18.2)	1.00		1.00	
25-40	25 (34.7)	141 (42.9)	0.62 (0.31, 1.24)	0.181	0.63 (0.27, 1.51)	0.308
≥41	30 (41.6)	127 (38.7)	0.83 (0.42, 1.62)	0.594	1.18 (0.471, 2.99)	0.714
Location						
Rural	26 (36.1)	27 (8.1)	1.00		1.00	
Urban	46 (63.8)	304 (91.8)	0.15 (0.08, 0.29)	0.001	0.15 (0.07, 0.31)	0.001
Method						
Drug	52 (74.2)	287 (88.5)	0.36 (0.13, 1.00)	0.052	0.28 (0.09, 0.88)	0.029
Poising	12 (17.1)	25 (7.7)	0.96 (0.28, 3.17)	0.947	0.61 (0.16, 2.35)	0.481
Other	6 (8.5)	12 (3.7)	1.00		1.00	
Marital status						
Other	7 (9.7)	37 (11.4)	1.00		1.00	
Married	38 (52.7)	153 (47.3)	1.31 (0.54, 3.17)	0.546	1.29 (0.49, 3.37)	0.600
Single	27 (37.5)	133 (41.1)	1.07 (0.43, 2.65)	0.879	0.85 (0.28, 2.54)	0.783
Literacy						
Illiteracy	11 (15.4)	18 (5.6)	1.00		1.00	
Subordinate-education	51 (71.8)	251 (79.1)	0.33 (0.14, 0.76)	0.008	0.70 (0.25, 1.97)	0.502
College education	9 (12.6)	48 (15.1)	0.30 (0.10, 0.86)	0.025	1.00 (0.27, 3.71)	0.993
Job						
Other	2(2.8)	14(4.4)	1.00		1.00	
Unemployed	39(54.9)	185(59.1)	1.47 (0.32, 6.75)	0.435	1.52 (0.30, 7.67)	0.610
Employed	30(42.2)	114(36.4)	1.84 (0.39, 8.55)	0.616	2.34 (0.45, 12.03)	0.306

## Discussion

In summary, our work represents the need to provide appropriate intervention in different parts of Hamadan Province to avert deaths and related injuries from attempted suicide. We observed two primary clusters in study areas, which represented only 17.87% of all cases and with the centrality of Asadabad and Razan. Underlying variables such as location, the method of action and literacy played a different role in a cluster and non-clusters areas. In other words, suicides by people who were rural, illiterate and non-drug user had more likely or odds to be in a cluster, similar to those of an Australian study^6^.

We observed the over-dosing method was the most common of suicides method in the cluster and non-cluster areas and there was a significant relationship between age and suicide methods (Chi_2_=11.32, *P*=0.023), which means that the distribution of cases tends to over-dosing as age increases. We also observed only 3.25% of peoples had under 15 yr old and most of them were between 25-40 yr, but some studies have reported high rates of suicides in adolescents^16-18^. Our results did not reveal a significant, sex difference between clusters and non-clusters and we did not observe a significant relationship between age and sex (*P*=0.050) similar to an Australian study^6^.

In keeping with previous studies ^16,19^, the over-dosing was the most common method of suicides in adolescents who attend hospital after their injury. However, there is a high heterogeneity in suicides methods and some studies^20,21^ had considered the self-cutting as the most common method, however difference subgroups of self- cutter most are not at risk for suicide or self-harms^17^. Some studies have shown that only a small proportion of suicides cases refer to a hospital or other routine health services ^18,22^ means that this behaviour is largely hidden at least for treatment centers ^16^, in other words, there is an iceberg pattern in societies. Probably this matter is ruling in our study and reported cases are only a small proportion of the total number of cases.

In contrast with our observation, various studies have shown the more common incidence of suicide in the female adolescents and its decreasing trend with age such as a European American adolescent’s study^23^. However, the main reasons for this phenomenon remain uncertain but more access to drugs, more deal with a stressful situation, more alcohol and drug consumption and social behaviors transition are likely involving factors^16^. Besides, in case of successful suicide, in more than of 30 Western countries rates in men are higher than in women ^24^. We did not observe a significant relationship between age and sex (*P*=0.05) similar to an Australian study^6^.

The broad classification of some variables and high missing percentage for some of their classes, undercounting of suicides cases due to social, individual and cultural reasons and stigmatization, direct referral of complete suicide cases to forensic medicine and failure to record their information as well as low sample size were the probable limitation of our study. However, despite noted limitation, this study is the unique spatial study with a multivariate approach in Hamadan Province based on pilot data.

We found that clusters and non-clusters areas had potential differences, we also mentioned the undercounting issues and iceberg phenomenon of suicide behaviors. Hence, to confronting against attempted suicide in Hamadan Province, we propose the use of appropriate, long-term and evidence-based education for high-risk‏ and vulnerable groups regarding the limitation of this study.

Finally, we suggest the implementation of the appropriate intervention in different parts of Hamadan Province to avert deaths and related injuries from attempted suicide, because the formation of clusters did not equal for all province and affected by several factors (some of them were considered in this study). However, other influencing factors such as unemployment^13^, income^13^, hopelessness^25^, anxiety disorders^26^, drugs, imitation from friends and family^27^, family problems and parental separation^28^, harassment and sexual problems could play important role in suicides behaviors and subsequently formation of clusters. Therefore, suicide and suicides behaviors are the complex phenomenon with multiple causes, that based on health sector reform program in Iran^29^, response to community needs such as suicides behaviors, need to the cooperation of other organizations beyond the health sector. Finally, our results can be used for the further individual investigation to obtain a‏ deep insight into involved factors in identified clusters.

## Conclusion

Finally, to prevent and control of suicides must take measures beyond the scope of health system and necessity to cooperation with other organs and institutions, especially families, as the most important and most influential organ on formation‏ and increase of suicide behaviors, (through the appropriate education programs). Because the most of health-related organizations perform routine and therapeutic measures after the occurrence of action and only for the small proportion of referred cases. On the other hand, further studies are needed based on the information obtained from the new registry system of suicide cases in Hamadan Province.

## Acknowledgements

This work has been supported by the Behavioral Disorders and Substance Abuse Research Center, Hamadan University of Medical Sciences, Hamadan, Iran.

## Conflict of interest statement

The authors declare that there is no conflict of interests.

## Funding

The study was funded by Vice-chancellor for Research and Technology, Hamadan University of Medical Sciences (No. 9507134139).

## Highlights

- Two significant clusters of attempted suicide were detected in Hamadan Province, western Iran with the centrality of Asadabad and Razan cities.
- Clusters and non-clusters areas were different in terms of location, self-harms methods and education.
- Rural areas residents, illiterate people and non-drug users had more likely to be in a cluster.
